# Optimization of Sugar-Derivatives Mixtures for Stabilizing Polyclonal Immunoglobulin G in Spray-Dried Inhalable Powders During Processing and Long-Term Storage

**DOI:** 10.3390/pharmaceutics18050573

**Published:** 2026-05-05

**Authors:** Philippe Gevenois, Le Van Bui, Thami Sebti, Yvan Vander Heyden, Karim Amighi, Nathalie Wauthoz

**Affiliations:** 1 Unit of Pharmaceutics and Biopharmaceutics, Université Libre de Bruxelles (ULB), 1050 Brussels, Belgiumle.bui@ulb.be (L.V.B.);; 2 Research and Development Department, Laboratoires S.M.B., 1080 Brussels, Belgium; 3 Department of Analytical Chemistry, Applied Chemometrics and Molecular Modelling, Vrije Universiteit Brussel (VUB), 1090 Jette, Belgium; yvan.vander.heyden@vub.be

**Keywords:** immunoglobulin G, inhalation, spray-drying, design of experiment

## Abstract

**Background/Objectives:** The development of dry powder formulations for pulmonary delivery of therapeutic antibodies requires careful stabilization strategies to preserve protein integrity during spray-drying and long-term storage. This study investigates the impact of various sugar-derivatives, a polyol (D-mannitol), a disaccharide (D-sucrose) and a polysaccharide (dextran 10 kDa), used individually or in combination, on the physical stability of bovine polyclonal immunoglobulin G (pAb) in dry powders for inhalation (DPIs). **Methods:** A design of experiments (DoE) approach was employed to evaluate the effects of these excipients on residual moisture (RM), low-order aggregates (LOA) and high-order aggregates (HOA), immediately after spray-drying (T0) and after 10 months of storage at room temperature in a desiccator (T10). **Results:** All DPIs exhibited a high amorphous content and a favorable glass transition temperature, with RM decreasing over time. The combination of D-mannitol and dextran 10 kDA (DPI-MD) demonstrated the most effective stabilization, minimizing LOA and HOA formation at T0 and T10. Although the ternary mixture, including D-sucrose (DPI-MSD) exhibited higher process stability, it was less stable over time in comparison to the binary mixture. The aerodynamic performance of these carrier-free DPIs, assessed via laser diffraction (% ˂ 5 µm), were between 51 ± 3 (DPI-MD) and 67 ± 4 (DPI MSD) and a Next Generation Impactor, confirmed that formulation produced aerosol with suitable size distribution and fine particle fractions (FPFn upt to 71 ± 5% for DPI-MSD), for deep pulmonary deposition. **Conclusions:** These findings highlight the importance of combining excipients with complementary physical properties to achieve robust protein stabilization. The DPI-MD emerged as the most promising candidate for pAb lung delivery, balancing protein integrity, powder stability, and aerodynamic efficiency.

## 1. Introduction

Since the pioneering development of the hybridoma technology by Kohler and Milstein in 1975 [1], monoclonal antibodies (mAbs) have gained importance as therapeutic agents because of their high affinity and specificity for target molecules/receptors [2]. Despite these advantages, therapeutic mAbs face several challenges, primarily due to their high-molecular weight (mw) and complex tertiary structure. Their production remains time-consuming and expensive, as it relies exclusively on biological systems [3], and the subsequent purification process often involves multiple steps [4]. Another major challenge lies in the structural instability of mAbs. Their tertiary structure is crucial for biological activity, but can be easily disrupted by external factors, such as heat, adsorption at air/water interfaces, or pH fluctuations for example [5]. Once unfolded, hydrophobic amino acid residues become exposed, promoting intermolecular interactions that can lead to aggregate formation [6]. These aggregates not only compromise therapeutic efficacy but also raise concerns regarding immunogenicity [7]. Aggregates can range from reversible low-order aggregates (LOA), such as dimers or trimers, to irreversible high-order aggregates (HOA), which are typically insoluble and often originate from LOA [8].

To limit aggregation and enhance protein stability, formulation strategies commonly employ stabilizing excipients. Moreover, transitioning proteins to a dry state offers several advantages over liquid formulations. Dry forms extend shelf life and eliminate water-mediated degradation pathways, such as deamidation or hydrolysis in the hinge region [9,10]. Removing 95–99% of the water also decreases transportation costs and permits ambient temperature shipping, in contrast to liquid protein formulations that often require cold-chain logistics due to limited long-term stability [9].

Among the scalable drying techniques, spray-drying offers notable benefits over freeze-drying (lyophilization), such as cost-effectiveness, speed, and the ability to engineer particles in a single step. Spray-drying transforms a liquid feed into dry particles, and various process parameters can be adjusted to influence particle size, shape, density, crystallinity, and residual solvent content [9,11]. This technique is particularly suitable for developing pharmaceutical formulations intended for non-injectable routes, such as pulmonary delivery [9,12].

Pulmonary administration via inhalation is a non-invasive route that enables direct drug delivery to the lungs, especially in the treatment of respiratory diseases. This localized delivery reduces systemic exposure and side effects, while maintaining therapeutic efficacy at lower doses. It also facilitates the delivery of hydrophilic and large molecules, such as proteins, to their site of action in the bronchial or alveolar lumen, bypassing biological barriers, such as endothelial and epithelial membranes encountered with systemic routes [12].

Dry powder inhalers are among the most common devices for pulmonary drug delivery. They are environmentally friendly and rely on the patient’s inspiratory airflow to aerosolize the powder [13]. However, the powder must possess suitable aerosolization and dispersion properties, which can be compromised by humidity [13]. Therefore, maintaining low and stable residual moisture (RM) is critical for long-term performance [12,14,15].

Currently, four protein-based dry powder formulations, produced via spray-drying, received Food and Drug Administration (FDA)-approval: Exubera^®^ Insulin (FDA approval 2006 but retrieved since 2007 [16]), Trelstar^®^ triptorelin pamoate (FDA approval in 2010 [17]), Somatuline^®^ lanreotide (FDA approval in 2007 [18]), Raplixa^®^ fibrin sealant (human) (FDA approval in 2015 [19]) and Inbrija^®^ levodopa (FDA approval in 2018 [20]).

While spray-drying can affect protein structure, the use of stabilizing excipients may help minimize degradation [12,21] during the three key steps of spray-drying: atomization, drying and particle separation from the gas phase. Among the stresses encountered during spray-drying, dehydration is particularly critical. Sugars and their derivatives are well-known for their protective role during protein dehydration and are commonly used as cryoprotectants in freeze-drying [22,23,24]. Although extensively employed in dry-state processes, the mechanisms underlying their stabilizing effects are still being elucidated. Furthermore, the impact of combining different sugars and derivatives with varying physical properties remains underexplored, especially in the context of spray-drying.

Therefore, this study aims to evaluate the effect of various sugar derivatives, each with distinct physical properties, on the stability of a model protein (bovine polyclonal IgG, pAb) following spray-drying and during long-term storage at room temperature (RT) in a desiccated environment. Storage in a desiccator was chosen since dry powders for inhalation are quite sensitive to humidity, as they can adsorb moisture on their surface which can increase capillary forces and change adhesion and therefore dispersion forces. Moreover, in case of amorphous content, moisture is adsorbed more tightly on an amorphous surface and can induce recrystallisation leading to change in adhesive forces and, therefore, during dispersion through inspiratory airflows. Dry powder for inhalation-based medicine often contains desiccant either inside the device close to the reservoir or in the primary container for capsule for example (12–15).

Protein stability in dry powder for inhalation (DPI) was assessed based on the residual moisture (RM) content, which impacts different physical and aerodynamic properties, and the contents of LOA and HOA, which are critical indicators of pAb stability. A rational mixture design of experiments (DoE) was employed to evaluate both the individual and interaction effects of sugar derivatives.

The selected excipients, D-mannitol (M), D-sucrose (S) and dextran 10 kDA (D), used as controllable input factors, were chosen based on their physical properties (such as mw, glass transition temperature (Tg) and hydrogen bonding potential) and their known tolerability in pulmonary applications:

1. D-mannitol (C_6_H_14_O_6_) is a small polyol (mw: 182.2 Da) with 12 potential sites of hydrogen-bonds (H-bonds) per molecule [25] (Equation (1)) and a very low Tg of 13 °C [26], which is authorized for inhalation by FDA [27].
2. Dextran 10 kDa (mw: 10,000 Da), a polysaccharide of D-glucose is a larger molecule with a very high Tg (213 °C) [22,28] and 310 potential H-bonds (Equation (2)). Moreover it showed promising results in terms of lung tolerability [29].
3. Because of the lack of lung toxicity data for non-reducing disaccharides, D-sucrose (C_12_H_22_O_11_), an inexpensive intermediate size sugar (mw 342.3 Da), which presents a very low oral toxicity, is widely used in both food and pharmaceutical industries, [30] and was tested. D-sucrose possesses 14 potential H-bonds per molecule [30] (Equation (1)) and an intermediate Tg (63 °C).

## 2. Materials and Methods

### 2.1. Material

pAb was obtained as a lyophilized powder from Equitech (Kerrville, TX, USA). Citric acid monohydrate and NaH_2_PO_4_ were purchased from Merck (Darmstadt, Germany). D-Sucrose (S) and L-Arginine were bought from Sigma-Aldrich (Saint-Louis, MI, USA). Trisodium citrate was purchased from Alfa Aesar (Haverhill, MA, USA). Dextran T10 (D) was obtained from Pharmacosmos (Holbaek, Denmark). Pearlitol 200SD-Mannitol (M) was purchased from Roquette (Lestrem, France). Na_2_HPO_4_ anhydrous, sodium hydroxide, sodium azide and silica gel were purchased from VWR Chemicals (Oud-Heverlee, Belgium). The bicinchoninic acid (BCA) protein assay kit, the microBCA kit and related globulin standard ampules were purchased from Thermofisher (Waltham, MA, USA). Millex polyvinylidene fluoride syringe filters, Durapore^®^, were purchased for Sigma-Aldrich. Bridged Ethylene Hybrid (BEH) size exclusion chromatography (SEC) standards were purchased from Waters (Antwerp, Belgium) or from Biorad (Temse, Belgium). The ultrapure water was produced with a Purelab system (Elga LabWater, Wycombe, UK).

### 2.2. Methods

#### 2.2.1. Calculation

- Theoretical calculation of the number of hydrogen bonds.

The calculation of the number of H-bonds for M and S, Equation (1) was used. For D, Equation (2) was used.

Equation (1): Calculation of the number of H-bonds which can possibly be formed by the unit of mass for D-mannitol and D-sucrose.

(1) $$H-bonds\;per\;unit\;of\;mass=\frac{H-bond\;donor+H-bond\;acceptor}{mw}$$

Define unit of mass and H-bond donors and H-bonds acceptors in the molecules.

Equation (2): Estimation of the number of H-bonds for dextran 10 kDa, based on 10,000 as the molecular weight (mw) and n being the number of anhydrous D-glucose units on Dextran 10 KDa. The H-bond per anhydrous glucose unit is 6 = m. The equation was back-tested on available D-glucose and oligosaccharides (up to n = 4) derivatives.

(2) $$Total\;of\;H-bonds=\frac{mw}{n}\times \left(m-1\right)=\frac{10,000}{162}\times 5$$

#### 2.2.2. Mixture Design of Experiments (DoE)

**Figure 1:**
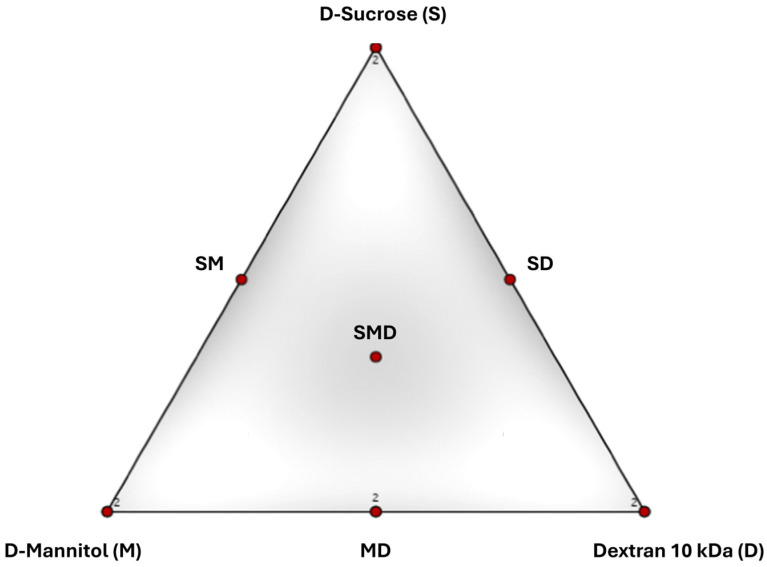
Simplex centroid design. Apexes represent individual excipients. The centroid and the middle of the axes represent a combination of 3 or 2 excipients, respectively. The 7 points represent the 7 formulations produced per design. (SD: 50% D-Sucrose, 50% Dextran 10 kDA; MD: 50% D-Mannitol, 50% Dextran 10 kDa; SM: 50% D-Sucrose, 50% D-Mannitol; SMD: 33% D-Sucrose, 33% D-Mannitol and 33% Dextran 10 kDa).

The DoE was a simplex-centroid mixture design (Figure 1). S, D and M were considered as controllable input factors. The other components (pAb and buffer species) were kept at constant concentration.

To assess pAb and powder stability, three responses were measured: the contents of HOA (%), LOA (%) and RM (%). The three responses were determined just after the spray-drying process (T0) and after a 10-month storage at RT in a desiccator with desiccant silica (T10). To model the response, a quadratic model was chosen following Equation (3). In practice, the three-factor interaction term (MSD) was not used in the model as it is generally considered negligible. Besides the individual responses (HOA, LOA, RM), also a global desirability value was determined according to the approach of Derringer and modeled.

Equation (3): Quadratic regression model used to fit the responses and to generate the response surfaces.

(3) $$y=\alpha_{1}S+\alpha_{2}M+\alpha_{3}D+\alpha_{1,2}MS+\alpha_{1,3}SD+\alpha_{2,3}MD+\alpha_{1,2,3}MSD$$

To calculate global desirability, a coefficient of importance was set for each individual response. The latter were first transformed into a 0 (undesirable) to 1 (most desirable) desirability scale. The coefficients of both LOA and HOA, which are both directly related to the stability of the pAB, were set to the highest importance included in the software (i.e., 5), while the coefficient of importance for RM, which is less directly related to the stability of the powder or the protein, but might be an indirect predictor, was set to 3. Global desirability is calculated as the geometric mean of the responses (desirability values) considering their importance. The global desirability plot, showing the response surface for desirability, was drawn. The software used for DoE design set-up and analysis was Design Expert V.12 (Statease, Minneapolis, MN, USA).

#### 2.2.3. pH Stability Evaluation of pAb

To determine the pH stability, 200 mg lyophilized pAb powder was dissolved in 10 mL of various 20 mM buffers either at pH 6.0 (i.e., citrate or L-histidine) or at pH 7.0 (i.e., citrate or phosphate) in 10 mL glass vials. The systems were left to equilibrate for 1 h at RT without stirring to ensure complete dissolution. The resulting solutions were placed for 24 h in a climatic chamber (Weiss Technic, Liedekerke, Belgium) set at 25 °C and 60%RH and then analyzed in terms of soluble and insoluble aggregates (LOA and HOA, respectively) as described in Section 2.2.6. Physical degradation—pAb aggregate determination in DPIs.

#### 2.2.4. Spray-Dried Dry Powder for Inhalation Formulations

The DPIs were produced with a mini-Spray-Dryer B-290 (Büchi, Flawil, Switzerland) in a single step. Briefly, pAb was dissolved during 1 h without stirring at a 10 mg/mL concentration in a 20 mM citrate buffer solution set at pH 7.0. This 50 mL-solution was supplemented with the different sugar derivative(s) (i.e., S, M and/or D) at a pAb/excipient weight ratio of 9:1 (Table 1). Then, this solution at 1.6% *w*/*v* of solid content (i.e., pAb, buffer and sugar derivatives) was pumped at a constant flow rate of 3 g/min and sprayed through a two-fluid nozzle (diameter 0.7 mm) via the nebulization gas (air), set at 820 L/h. This sprayed solution is dried into dry powder thanks to an inlet temperature set at 110 °C (leading to an outlet temperature between 55 and 60 °C) and the dry powder was then collected through a high-performance cyclone with an aspiration flow rate set at 35 m^3^/h. The process yield is calculated using Equation (4).

Equation (4): Relationship to calculate the yield from spray-drying.

(4) $$Yield\left(\%\right)=\left(100-residual\;moisture\;of\;collected\;powder\left(\%\right)\right)*(\frac{mass\;of\;collected\;powder\left(g\right)}{\sum mass\;of\;solid\;components\;in\;solution\left(g\right)})$$

The DPIs were analyzed just after the spray-drying process (T0) and then stored at RT in plastic recipients enclosed in a desiccator, containing silica desiccant, for up to 10 months (T10).

#### 2.2.5. Physicochemical Properties of DPIs

- Morphology—Scanning Electron Microscopy (SEM).

The DPIs were visualized by SEM using a Hitachi SU-70 ultra-high-resolution microscope (Hitachi, Tokyo, Japan). The particles were coated with gold (35 mA for 4.5 min at 1 mbar under argon) before analysis. The acceleration voltage during the observation was 20 kV. These analyses were outsourced to the 4MAT lab (Université Libre de Bruxelles, Ixelles, Belgium). Analyses were made in monoplicate.

- Residual moisture—Thermogravimetric Analyses.

RM within the DPIs was assessed using a thermogravimetric analyzer Q500 (TA Instruments, New Castle, DE, USA). Briefly, about 10 mg sample was loaded on a platinum pan (TA Instruments) and heated from RT to 200 °C at a constant heating rate of 10 °C/min under nitrogen atmosphere. Data acquisition was performed using the TA advantage software (version 5.5.24) and the data analysis with the TA instruments Trios software (version 4.5.0.42498). RM in the samples was attributed to the sample weight loss between RT and 150 °C. TGA analyses were performed at T0, T6 and T10 in monoplicate.

- Glass transition temperature—Modulated Differential Scanning Calorimetry (MDSC).

The thermal transition events of the DPIs were analyzed with MDSC, using a modulated differential scanning calorimeter Q200 instrument equipped with a RCS90 cooling system (TA Instruments). About 3.0 to 5.0 mg pAb or DPIs were accurately weighted into a Tzero aluminum pan (TA Instruments), which was sealed with a hermetic lid (TA Instruments). An empty Tzero aluminum pan, sealed with a hermetic lid, was used as the reference. The sample and reference pans were then simultaneously submitted to three cycles performed under N_2_: cycle 1: heated from −50 °C to 125 °C at a heating rate of 10 °C/min; cycle 2: quenched to −50 °C, and finally; cycle 3: reheated from −50 °C to 125 °C at a heating rate of 10 °C/min.

Alternatively, MDSC was used to separate kinetic from thermodynamic events. The sample and reference pans were then simultaneously submitted to one cycle performed under N_2_ where they were heated from −50 °C to 100 °C using an average heating rate of 3.0 °C/min, a modulation temperature amplitude of ± 0.8 °C and a period of 40 s. Data acquisition was performed using the TA Advantage software (version 5.5.24) and analysis with the TA instruments Trios^®^ software (version 4.5.0.42498). The Tg was determined as the midpoint of the transition. Samples were analyzed at T10.

- Crystalline properties—X-Ray Powder Diffraction (XRPD).

The crystalline/amorphous structure of the DPIs was analyzed using an X-Ray diffractometer (D8 Advance Eco Bruker, Madison, WI, USA) equipped with a one-dimensional silicon detector (LynxEye, Bruker AXS) using Cu Kα radiation (1.54 Å; 40 kV × 25 mA). The angular range was set at 3–45° 2θ with a step size of 0.02° and a dwell time of 1 s. Analyses, made in monoplicate, were outsourced to the 4MAT lab (Université Libre de Bruxelles).

#### 2.2.6. Physical Degradation—pAb Aggregate Determination in DPIs

- Sample preparation.

The quantity of DPIs containing approximately 10 mg pAb were accurately weighted in 2 mL protein LoBind microcentrifuge tubes (VWR, Oud-Heverlee, Belgium) and resuspended in the proper volume of phosphate-buffered saline (PBS) pH 7.4 solution to obtain a 10 mg/mL solution. Each tube was closed, inverted several times, and briefly vortexed (˂5 s) to ensure proper resuspension of the powder. The tubes were then briefly centrifugated (2000× *g*) using a MiniStar microcentrifuge (VWR) and incubated at RT for about 30 min. The solution/suspension of reconstituted powder was then briefly vortexed for proper homogenization and diluted 10-fold. Approximately 80% of the diluted solution was collected using a 1 mL polypropylene syringe (VWR) and filtered on 0.22 µm 13 mm polyvinylidene difluoride (low-protein binding) syringe filters (Sigma-Aldrich) to remove HOA. The HOA are defined by the filter pores size, namely the fraction above 220 nm. The remaining 20% of the diluted solution was kept unfiltered to determine insoluble aggregates percentages. Each sample was prepared in triplicate.

- Determination of high-order aggregates percentage and soluble protein recovery.

20 µL of each filtered (containing pAb and LOA) and non-filtered solution/suspension (containing pAb, LOA and HOA) were analyzed using the Pierce™ BCA protein assay kit according to the procedure described in the instructions provided by the supplier. Briefly, a 7-point pAb standard calibration curve from 125 to 2000 µg/mL was prepared from a commercial 2 mg/mL standard of bovine IgG. In total, 20 µL was pipetted three times into a 96-well microplate. A blank (dilution buffer-PBS at pH 7.4) was pipetted at least in triplicate. Then, 20 µL of the filtered and non-filtered reconstituted solutions/suspensions were then pipetted in triplicate using a 20 µL single channel Finnpipette F1 (ThermoFisher Scientific, Merelbeke, Belgium). The two reagents A (BCA-containing reagent) and B (CuSO_4_ reagent) were then mixed at a ratio of 50:1 (A:B) *v*/*v* and 200 µL was added to each well containing the standards, the blanks and the samples using a 8-channel research plus variable pipette (Eppendorf, Aarchot, Belgium). The plate was covered with a film plate sealer and incubated at 37 °C for 30 min, while avoiding light exposure. Subsequently, the plate was read using a Multiskan^®^ FC plate reader at 570 nm (ThermoFisher Scientific). The average absorbance of the blanks was subtracted from the standard and sample absorbances, and the concentrations of the samples were interpolated from the standard curve using a second order polynomial regression model. Analyses were made in triplicate. The insoluble aggregates are thereby defined by the filter pores size, namely the fraction above 220 nm. The HOA percentage was estimated using Equation (5) and the soluble protein recovery (%) using Equation (6).

Equation (5): High-order aggregates (HOA) content (%).

(5) $$HOA\left(\%\right)=100\times \frac{Protein\;concentration\;of\;unfiltered\;solution-Protein\;concentration\;of\;filtered\;solution}{Proteincon\;centration\;of\;unfiltered\;solution}$$

Equation (6): Soluble protein recovery (%).

(6) $$Soluble\;protein\;recovery\left(\%\right)=100\times \frac{Protein\;concentration\;in\;filtered\;solution}{Protein\;concentration\;in\;unfiltered\;solution}=100-HOA\left(\%\right)$$

- Determination of monomer content—semi-quantitative analysis.

The absolute total protein concentrations of the filtered solutions were first analyzed by UV-Visible Spectrophotometry at 280 nm with 320 nm used as reference with a nanophotometer NP80 (Implen, Munich, Germany). Measurements were made using an ultra-micro quartz cuvette (Hellma, Müllheim, Germany). Prior to the analysis, a blank, corresponding to PBS at pH 7.4, was analyzed, and automatically subtracted from each of the other measurements by the NP80’s acquisition software. A one-concentration standard curve was analyzed to interpolate the sample’s concentrations.

- Determination of low-order aggregates percentage.

Prior to the SEC samples, a protein standard with known mw was analyzed to assess the column efficacy and to estimate its elution time. A 20 µL sample, prepared from filtered solution, was then injected into a high-pressure liquid chromatography system (Agilent Technologies, Santa Clara, CA, USA) equipped with a degasser, a quaternary pump, a thermostat oven fixed to 25 °C and a diode array detector fixed to 280 nm and 600 nm as working and reference wavelengths, respectively. The analyte was eluted at 1 mL/min for 15 min with a 170 mM phosphate buffer pH 6.8 supplemented with 200 mM L-Arginine, through a XBridge Protein BEH SEC column (200 Å, 3.5 µm, 7.8 mm × 300 mm) connected to its guard column (30 mm) (Waters, Milford, CT, USA). Upon completion, the mobile phase was filtered on Nalgene 0.2 µm polyethersulfone membrane (ThermoFisher Scientific) and then autoclaved (121 °C, 15 min) to reduce microbial load.

The low-order aggregates (LOA) content (%) was estimated using Equation (7) from the sum of area under the curve (AUC) of all peaks eluting before the monomer peak, which corresponds to pAb, divided by the sum of all AUC peaks (from SEC analysis). All samples were determined in triplicate.

Equation (7) determines low-order aggregates—soluble protein fraction after sample filtration step eliminating high-order aggregates.

(7) $$Low\;order\;aggregates\left(\%\right)=100\times \frac{\sum {AUC}_{before\;eluting\;monomer\;peak}}{{AUC}_{total}}(\%)$$

Monomer recovery (%) was determined using Equation (8) using the AUC from the monomer in SEC and the theoretical AUC calculated from the standard curve based on the soluble protein concentration precisely determined by UV spectrophotometry.

Equation (8) determines monomer recovery (%) after sample filtration step to eliminate high-order aggregates.

(8) $$Monomer\;recovery\;(\%)=100\times \frac{{AUC}_{monomer}measured}{{AUC}_{monomer}theoretical}$$

#### 2.2.7. Aerosolization and Dispersion of DPIs Through Dry Powder Inhaler

The size distribution of aerosol particles, generated from each DPI through a low resistance Axahaler^®^ inhaler (S.M.B. Laboratories, Brussels, Belgium), was first determined with a laser-based diffraction technique, Spraytec (Malvern Analytical, Worcestershire, UK), that is equipped with an inhalation cell, specifically modified for measuring the particle size diameter (PSD) generated from medicinal aerosols, such as Metered Dose Inhalers, Dry Powder Inhalers and Nebulizers. This technique has demonstrated a good correlation with cascade impactor analysis for carrier-free DPIs [31]. Briefly, approximately 20 mg DPI was weighed into a size 3 hydromellose capsule (Qualicaps, Madrid, Spain), placed into an Axahaler^®^ capsule-based inhaler and connected—using the appropriate mouth adaptor—to the induction port of the cascade impactor from the Multi-Stage Liquid Impactor, fixed on the closed mode Spraytec. Then, a critical air flow (100 L/min) was applied for 2.4 s using two HCP5 pumps connected to a TPK2000 flow controller (Copley Scientific, Nottingham, UK). The flow was controlled prior to the test using a DFM3 flow meter (Copley Scientific). The triggering mode was set at 10%, the data acquisition rate to 2500 Hz, the acquisition duty cycle at 50%, the test duration at 3000 ms, and the refractive index at 1.50 (for standard opaque particles). The data acquisition was made using the RTsizer software 5.51 (Malvern Analytical) and the dgeo distribution parameter was extracted as the volume-weighted mean diameter [D4,3], the median diameter (d0.5), representing the diameter of 50% of the cumulative volume of the particles), and the diameter of 90% of the cumulative volume of the particles (d0.9). The percentage of particle inferior to 5 µm was also extracted as it is generally correlated to FPF from carrier free DPI [31]. The analyses were made in triplicate.

Then, the aerodynamic behavior of the DPI, which showed the best result through the laser diffraction-based technique and one of the best desirability results through the DoE, was assessed using the Next Generation Impactor (NGI, Copley Scientific). Briefly, 20 mg DPI was weighed into a size 3 hypromellose capsule (Quali-caps) and placed into an Axahaler^®^ inhaler. The inhalation device was connected to the induction port of the impactor using the appropriate adaptor and a critical air flow (100 L/min) was applied for 2.4 s using two HCP5 pumps connected to a TPK2000 flow controller. After the deposition, each stage of the impactor (including the inhalation device and its adaptor, the induction port, and the pre-separator) collections were made with PBS solution in volumetric flasks and analyzed using a microBCA protein assay. Briefly, a calibration curve was constructed from 2 to 200 µg/mL using the BGG standard; the volume of standards and calibrator used in the test was 150 µL (instead of 20 µL); and the detection reagent included a third proprietary reagent solution (mix of reagents of the kit = 25A:24B:1C *v*/*v*/*v*). The test was run in triplicate. The median mass aerodynamic diameter (MMAD) and the geometric standard deviation (GSD) were calculated with the Copley inhaler testing data analysis software (Copley Scientific). The fine particle dose was reported relative to the dose of pAb in the capsule resulting in the fine particle fraction related to the nominal dose (FPFn). Another DPI was chosen arbitrary to evaluate the correlation between % inferior 5 µm obtained by laser diffraction technique and the FPFn obtained by using NGI.

#### 2.2.8. Statistical Analysis

Variance homoscedasticity was assessed with the Brown-Forsythe test. Data were then analyzed using two-way analysis of variance (ANOVA) with an α value set at 0.05. When a *p*-value below 0.05 was observed, the ANOVA was significant and a post hoc test, such as Tukey’s multiple comparison test, was applied for time or formulation analysis. The analyses were performed with 39 (GraphPad, San Diego, CA, USA).

## 3. Results

Initially, a DoE approach was employed to assess the impact of the sugar derivatives—used individually or in combination—primarily on the pAb stability, focusing on both LOA and HOA formation, and secondarily on the RM content of the DPIs, immediately after spray-drying (T0) and after 10 months of storage in a desiccator with silica gel (T10). To gain a deeper insight into the underlying stabilization mechanisms, the glass transition temperature (Tg) of the DPIs was also determined. As the DPIs are intended to be delivered by inhalation, aerosol particle size distribution was also assessed for all DPIs. Finally, the aerodynamic performances of two DPIs were assessed, one based on their desirability and PSD and another chosen arbitrary.

### 3.1. Stable-Buffer Determination for pAb

**Figure 2:**
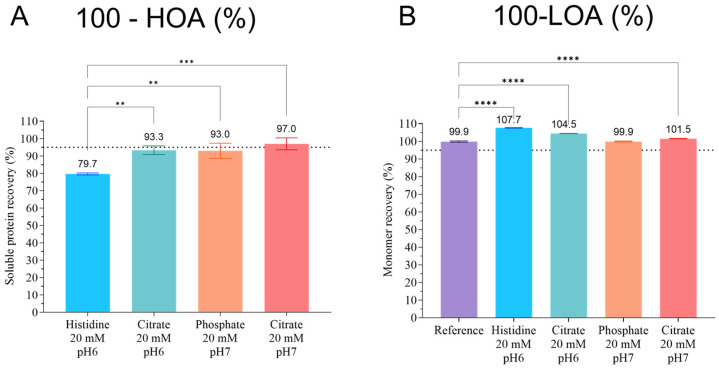
(**A**) Soluble protein recovery (%) measured by the bicinchoninic acid assay, and (**B**) monomer recovery obtained by size-exclusion chromatography for the buffer study (type, pH, concentration). Results are expressed as the mean ± standard deviation (*n* = 3). Statistical analysis was performed using one-way ANOVA and the post hoc Tukey’s test. ** = *p* < 0.01, *** = *p* < 0.001, and **** = *p* < 0.0001. The reference for the monomer recovery is the non-formulated monoclonal antibody raw material. The dashed line refers to 95% (i.e. max 5% of degradation).

A preliminary buffer screening was conducted to identify the optimal solution conditions for pAb, prior to the addition of sugar derivatives for the spray-drying process. To this end, the short-term stability was evaluated over 24 h at 25 °C across various pH-buffered environments. Among the tested conditions (Figure 2), citrate buffer 20 mM at pH 7.0 yielded the highest soluble protein content (97 ± 4%), while L-histidine buffer resulted in the lowest (79.7 ± 0.6%), with statistically significant differences observed (*p* ˂ 0.01 vs. citrate pH 6.0 and phosphate pH 7.0; *p* ˂ 0.001 vs. citrate pH 7.0, Tukey’s test). Monomer recoveries (%) were all not lower than 100%. All comparisons were statistically significant due to the small variability values (*p* ˂ 0.0001, Tukey’s test), but were not relevant from a practical point of view (Figure 2). Citrate buffer (20 mM, pH 7.0) was selected for the subsequent formulation steps because of its minimal formation of insoluble HOA, which is critical for both therapeutic efficacy and immunogenicity. Additionally, monomer recovery was not lower than 100%.

### 3.2. Dry Powders for Inhalation Produced by Spray-Drying

In addition to buffer agents, various excipients are commonly incorporated into liquid protein formulations intended for drying, to mitigate protein degradation. Sugar and sugar derivative as well as polyol are well known to mitigate the protein degradation during freeze-drying and were, have been or will be evaluated in spray-drying process [8,9,12].

Carbohydrates are the excipients of choice, when formulating protein pharmaceuticals due their propensity to form a glass matrix upon drying and to make multiple H-bonds [12], making them particularly effective to protect protein upon dehydration. However, due to their reducing ability, monosaccharides cannot be used as such as they would lead to glycation. Therefore, only derivatives of monosaccharides (i.e., polyol) are used for this purpose. Polyols have a high potential for hydrogen bonding, which plays a major role in the water-replacement protein stabilization mechanism during drying [12]. Furthermore, it has been proposed that, besides their H-bond ability, small saccharides can fill the free volume of the protein, thereby reducing the local mobility (β-motion) and increasing the stability despite their lower Tg [32]. D-mannitol is currently the only monosaccharide derivative used for FDA-approved inhaled therapeutics [33] and numerous examples of its successful use as a stabilizer can be found in the literature [12,21] although its crystallization during processing has shown the potential to deleteriously affect product stability [21]. Disaccharides are the most used carbohydrates when formulating protein pharmaceuticals for spray-drying (50%) [21]. Reducing sugars, such as D-lactose, which is the only disaccharides approved by the FDA for the pulmonary route [33], are described in the literature for protein stabilization, their use should be avoided to avoid Maillard reaction [14]. In addition, non-reducing sugar such as D-trehalose and D-sucrose are massively investigated [12,21]. In terms of spray-drying, D-trehalose seems to be more successful than D-sucrose because it has been suggested that the former presents superior abilities to form an H-bond with proteins and produce formulations with a higher Tg [21]; however, contradictory results were also reported as D-sucrose was reported to better fill protein free volume [34]. Polysaccharides are commonly used in the formulation of protein pharmaceuticals due to their a high Tg [12] by nature and their propensity to form glass matrixes [21]. Dextran 10 kDa [35,36] have shown interesting aerosolization properties. Dextran 10 kDa has also shown a good tolerance profile in Beagle dogs 473,476. In contrast, polyols usually have a quite low Tg [26,37] but they may have useful aerosolization properties [14] and have been shown to stabilize biological molecules efficiently [38].

This study focused on characterizing the effects of three well-known excipients: a polyol (M), a disaccharide (S), and a polysaccharide (D). The investigation aimed to evaluate not only the individual effects of each excipient, but also their interactions, to better understand their combined influence on pAb stability. All DPI were successfully formulated with a high yield of powder, between 71.6% (DPI based on MD) and 79.4% (DPI based on SD), recovered from the theoretical anhydrous value (Table 1).

#### 3.2.1. Physicochemical Properties—Morphology, Residual Moisture (RM) and Glass Transition Temperature (Tg) of DPIs

**Figure 3:**
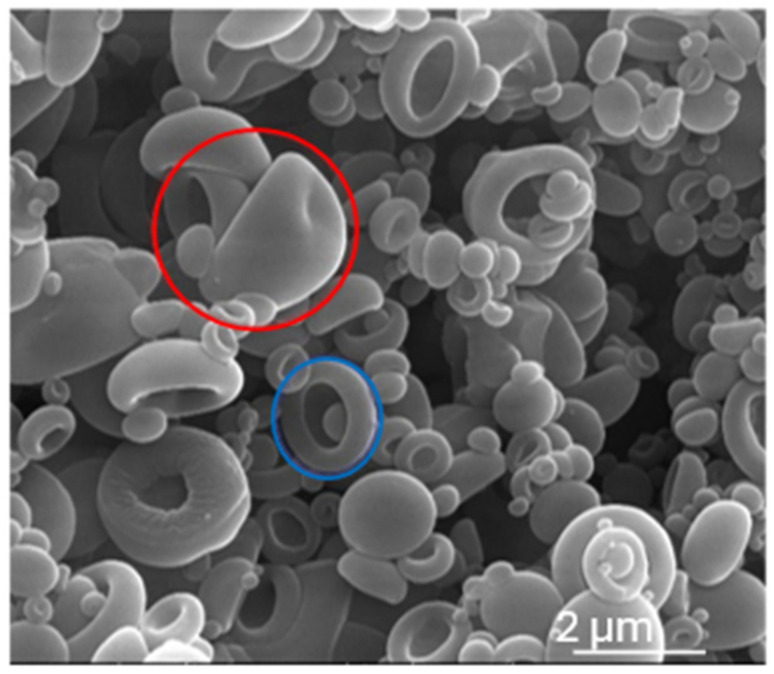
Particle morphology image obtained using scanning electron microscopy (magnification 24.000×) for the spray-dried formulation with sucrose and dextran 10 kDa, but representative for all formulations. Particles have a smooth, dimpled shape (red circle) or a doughnut shape (blue circle).

As observed in the SEM images, the spray-dried powder particles are around 2 µm and showed a smooth surface with a dimpled or a doughnut shape (encircled in red and in blue in Figure 3, respectively).

**Figure 4:**
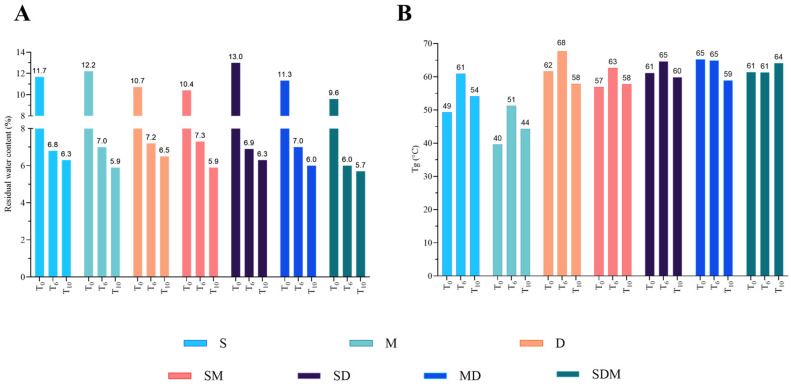
(**A**) Residual moisture obtained during thermogravimetric analysis on 10–20 mg powder samples after spray-drying (T0), after 6 months (T6) and after 10 months (T10). (**B**) Glass transition temperature (Tg) of the powders after 10 months, obtained using modulated differential scanning calorimetry. Formulations were stored at room temperature in a desiccator with silica gel (*n* = 1 for all experiments).

The results of RM and Tg for the DPI produced by spray-drying are reported in Figure 4.

All DPIs showed a high amount of RM at T0 (between 9.6% for DPI-MSD and 13.0% for DPI-SD), which decreased upon storage in a desiccator with silica desiccant for 10 months (T10), towards a stagnation comprised between 5.7% (DPI-MSD) and 6.5% (DPI-D) (Figure 4A).

MDSC was not assessed beyond 125 °C because of decomposition of the samples above 160 °C. All MDSC thermograms showed a single Tg and no other thermal event until 125 °C, indicating a remaining monophasic amorphous dispersion of the DPIs, even after 10 months of storage at RT with desiccant. DPIs containing D showed the highest Tg, which was maximal for DPI-MSD (64.1 °C), while the lowest was measured for the DPI-M (44.3 °C) (Figure 4B).

#### 3.2.2. XRPD on DPIs

**Figure 5:**
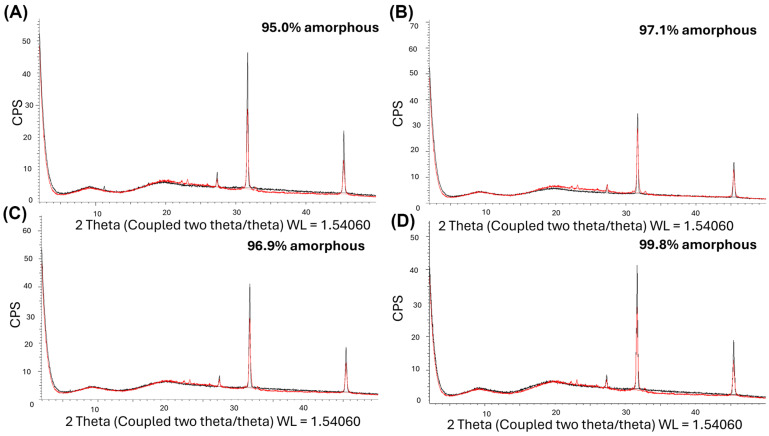
Example of X-ray diffraction on powder and amorphous percentage obtained by X-ray diffraction for the spray-dried formulations of polyclonal antibody generated at T0 (**A**–**D**); *n* = 1. The Black curves represent a formulation with (**A**) no stabilizer, (**B**) D-sucrose, (**C**) MSD, and (**D**) dextran 10 kDa. The spectrum of the raw material, containing the polyclonal antibody, is overlaid in red. Amorphous content is expressed relative to raw material content.

Crystallinity of the DPIs was evaluated using X-ray diffractometry. At T0 (Figure 5A–D), all DPIs showed a similar amount of amorphous content, between 95.0% (DPI with no stabilizer) and 99.8% (DPI with Dextran 10 kDa). The amorphous matrix demonstrated during MDSC is confirmed by the XRPD diffractograms. The same three peaks (2θ = 27, 32, and 45) were observed in each DPI as well as in the pAb raw material, which therefore certainly correspond to the small amount of phosphate buffer in the pAb raw material or to other impurities.

#### 3.2.3. pAb Aggregates Determination in DPIs

The pAb stability was evaluated by the determination of the LOA and HOA contents (%) in the DPIs and reported in Table 2.

The pAb resisted well to the formation of insoluble aggregates (HOA) during the spray-drying process as the HOA content remained very low at T0 (between 7 ± 2% for DPI-S and negligible with 1 ± 5% for DPI-M or 1 ± 1% for DPI-D, Table 2). The DPIs were not different from one another, and no significant degradation occurred over time (*p* > 0.05, two-way ANOVA).

The soluble aggregates (LOA) were of the same order of magnitude as the HOA after the spray-drying, i.e., between 4 ± 2% for DPI-S and negligible (−0.6 ± 0.6%) for DPI-MSD (Table 2). Here, statistically significant differences were observed between the DPIs (*p* < 0.0001, two-way ANOVA) and overtime (*p* < 0.0001, two-way ANOVA) with a significant interaction between formulation and time (*p* < 0.001, two-way ANOVA).

At T0 (Table 2), combinations with D-Mannitol and dextran 10 kDa were very efficient to stabilize pAb as DPIs based on MD and MSD had the LOA content considered as negligible at T0 (0.2 ± 0.4% and −0.6 ± 0.6%, respectively). The DPI based on MD had a significantly lower and negligible LOA content than the DPIs with a sugar or polyol individually (negligible (0.2 ± 0.4%) for DPI-MD versus 4 ± 2 for DPI-S, *p* < 0.0001; 2.3 ± 0.9% for DPI-M, *p* < 0.05; or 2.3 ± 0.4% for DPI-D, *p* < 0.05; Tukey’s test). The DPI-MSD was even more effective with negligible LOA content just after the spray-drying process (−0.6 ± 0.6% for DPI-MSD vs. DPI-S, *p* < 0.0001; vs. DPI-M or DPI-D, *p* < 0.001; and vs. DPI based on MS, *p* < 0.05; Tukey’s test). On the other hand, the least efficient excipient at T0 was D-sucrose as it showed the highest LOA content (4 ± 2%).

The tendency remained the same after 10 months storage in a desiccator at RT (T10), except that the differences between the DPIs were reduced. D-sucrose was still the least effective excipient (4.07 ± 0.06%) compared to some DPIs containing sugar/polyol combinations (2.0 ± 0.3% for DPI-MS, *p* < 0.05 or 1.5 ± 0.3% for DPI-MD, *p* < 0.01; Tukey’s test) but was not less effective than DPI-D (5.2 ± 0.2%) or DPI-M (3.5 ± 0.6%) used alone, or DPI-SD (2.8 ± 0.32%) or DPI-MSD (3.6 ± 1.2%) (*p* > 0.05 for both, Tukey’s test). At 10 months, the association of MD in DPI became more effective than that of MSD (1.5 ± 0.3% for DPI-MD versus 3.6 ± 1.2% for DPI-MSD, *p* < 0.05; Tukey’s test) and remained more effective than all excipients used individually (4.07 ± 0.06% for DPI-S, *p* < 0.01, 3.5 ± 0.6% for DPI-M, *p* < 0.05; and 5.2 ± 0.2% for DPI-D, *p* < 0.0001, respectively; Tukey’s test).

Although, the LOA content in DPI-MD was still lower (1.5 ± 0.3%) than DPI-MS (2.0 ± 0.3%) and DPI-SD (2.8 ± 0.3%), the difference was not statistically significant (*p* > 0.05, Tukey’s test). On the opposite, DPI-MSD (3.6 ± 1.2%) was neither more effective than DPI-S (4.07 ± 0.06; *p* > 0.05, Tukey’s test) and DPI-M (3.5 ± 0.6%; *p* > 0.05, Tukey’s test) nor than DPI-SD (1.8 ± 0.2; *p* > 0.05, Tukey’s test) and became less effective than DPI-MS (2.0 ± 0.3%; *p* < 0.05, Tukey’s test). Dextran 10 kDa (5.2 ± 0.2% for DPI-D) became the least protective excipient as, even though the difference with D-sucrose (4.07 ± 0.06% for DPI-S) was not statistically significant (*p* > 0.05, Tukey’s test). DPI-D had the highest LOA content (5.2 ± 0.2%, *p* < 0.0001, *p* < 0.01, *p* < 0.001 vs. MD, SD, SM, respectively, Tukey’s test).

Associating excipients, especially associations of D-mannitol and dextran 10 kDa, stabilized the pAb more efficiently than when used individually. Adding D-sucrose to the association MD, positively impacted the stability at T0, but somewhat destabilized pAb over time. Therefore, the best combination for stabilization during the spray-drying process and over time, was found with DPI-MD for LOA (0.2 ± 0.4% at T0 and 1.5 ± 0.3% at T10 for DPI-MD) which is better than DPI-MSD for LOA (−0.6 ± 0.6% at T0 and 3.6 ± 1.2% at T10 for DPI-MSD). HOA was negligeable and considered null at T0 and T10.

#### 3.2.4. Design of Experiments Analysis

To predict what formulation composition would give the best compromise between (i) the lowest RM; (ii) the lowest LAO content; and (iii) the lowest HAO content at T0 and/or T10, each response—as well as the global desirability—were modeled (Design Expert software) using a quadratic model (Equation (3)).

**Figure 6:**
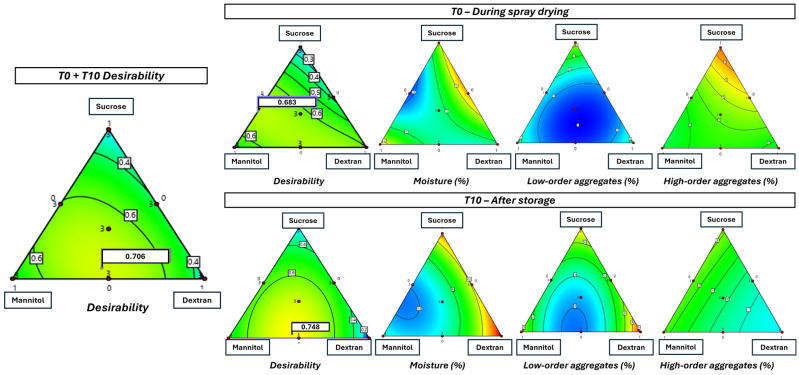
Contour plots obtained from modeling the individual responses and the desirability at T0 or T10. Desirability of the response is shown as a color gradient from blue (less desirable) to red (most desirable).

A contour plot was then drawn for each response and the global desirability (Figure 6). According to the model, the results converged towards an optimal zone encompassing a binary mixture with about equal fractions of D-mannitol and dextran 10 kDa, towards a ternary mixture with the addition of a low fraction of D-Sucrose.

Still according to the models, the optimal formulation at T0 with a desirability of 0.683 would comprise a proportion of 64% D-mannitol combined with 36% D-sucrose and 0% dextran 10 kDa or with a desirability of 0.683 would comprise a proportion of 38% of D-mannitol combined with 61% of dextran 10 kDA. However, for this short term protein stability, the binary mixture with about equal fractions (50/50 *w*/*w*) of D-mannitol and dextran 10 kDa also provides a high desirability value (0.665) (Figure 6).

For long-term stability, e.g., at T10, reducing D-sucrose and increasing Dextran 10kDA in the formulations is more beneficial, with the ternary mixture of 52.6% D-mannitol, 43.5% dextran 10 kDa and 3.9% D-sucrose, or the binary composition 54.7% D-mannitol and 45.3% Dextran 10kDa, presenting both the highest desirability (0.748). Again, the binary mixture with equal amounts of D-mannitol and dextran 10kDa provides similarly high desirability values (0.744).

When considering both short and long-term stabilities, i.e., T0 and T10, the excipients mixture 49.9% D-mannitol, 42.9% dextran 10 kDa and 7.1% D-sucrose showed the highest desirability (0.706). The binary composition 51% D-mannitol and 49% dextran 10 kDa presented a comparably high desirability of 0.704. Given the facts that a simpler mixture with two compounds is preferred to a more complex one with three, and that the results for the composition 50% D-mannitol and 50% dextran 10kDa are available and quite similar in terms of desirability (0.703), no additional experiments at predicted optima were performed.

The DOE performed on raw data revealed that the formulation composition significantly affected moisture and LOA at both T0 and T10 (Table 3). These responses were well described by quadratic mixture models, with strong effects between D-mannitol, D-sucrose, and/or dextran 10 kDa. In contrast, HOA exhibited high experimental variability and could not be reliably modeled, particularly at T10 as shown by a significant lack of fit (Table 3).

The quadratic model was suggested for moisture at T0 and T10 as well as for T0 LOA. Therefore, the quadratic was chosen as model for which an analysis of variance (ANOVA) was performed.

The quadratic regression model equation used is reported in Equation (3). The corresponding ANOVA results describing the effects of D-sucrose, D-mannitol, and dextran 10 kDa on the response are presented in Table 3 using the quadratic model.

For moisture and LOA measured at T0 and T10, the quadratic models were statistically significant (*p* ˂ 0.05) (Table 3). At T0, the moisture was strongly influenced by D-mannitol and D-sucrose (*p* = 0.0002), and to a lesser extent by the D-mannitol and dextran 10 kDa (*p* = 0.0266). The quadratic model was acceptable and predictive. At T10, moisture was strongly dependent on formulation composition, and the model exhibited high robustness and predictive ability.

The terms SM, SD, MD do not stand for interaction effects because they are also affected by the quadratic terms.

For instance, for mixture variables the quadratic term from a regular model corresponds to Equation (9). Thus, in a canonical representation the quadratic term affects the coefficients for S, SM, SD. Similarly, the other quadratic terms can be considered. Therefore, the discussion of individual terms and interactions does not make a lot of sense from a practical perspective.

(9) $$S^{2}=S\times S=S\times \left(1-M-D\right)=S-SM-SD$$

At T0, LOA strongly depended on formulation composition. The model was considered solid and exploitable. At T10, the LOA model showed excellent predictive performance; however, the presence of lack of fit (*p* = 0.003) suggested that caution should be exercised when interpreting results.

For HOA, the quadratic model was not significant at T0 (*p* = 0.0718). At T10, the HOA model was also not significant (*p* = 0.2017) and exhibited high experimental variability. Consequently, HOA could not be reliably modeled at either T0 or T10.

#### 3.2.5. Aerodynamic Behavior of DPIs

- Aerosol particle size distribution—Laser diffraction technique.

**Figure 7:**
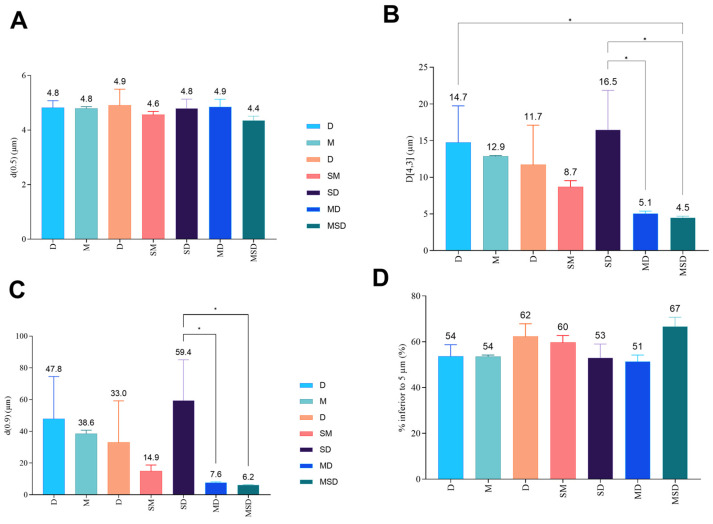
Aerosol particle size distribution parameters using SprayTec laser diffraction including (**A**) the median diameter d(0.5), (**B**) the diameter at 90% of the particle size distribution d(0.9), (**C**) the volume-weighted-mean diameter D[4,3] and (**D**) the percentage of particles with a diameter inferior to 5 µm. The Axahaler^®^ device was filled with a hypromellose capsule containing 20 mg polyclonal DPIs. Mean ± standard deviation, *n* = 3. Statistical analysis was performed using one-way ANOVA followed by Tukey’s test. Symbols are defined as * for *p* < 0.05. MS, D-mannitol/D-sucrose; SD, D-sucrose/dextran 10 kDa; MD, D-mannitol/ dextran 10 kDa; MSD, D-mannitol/D-sucrose/dextran 10 kDa.

**Figure 8:**
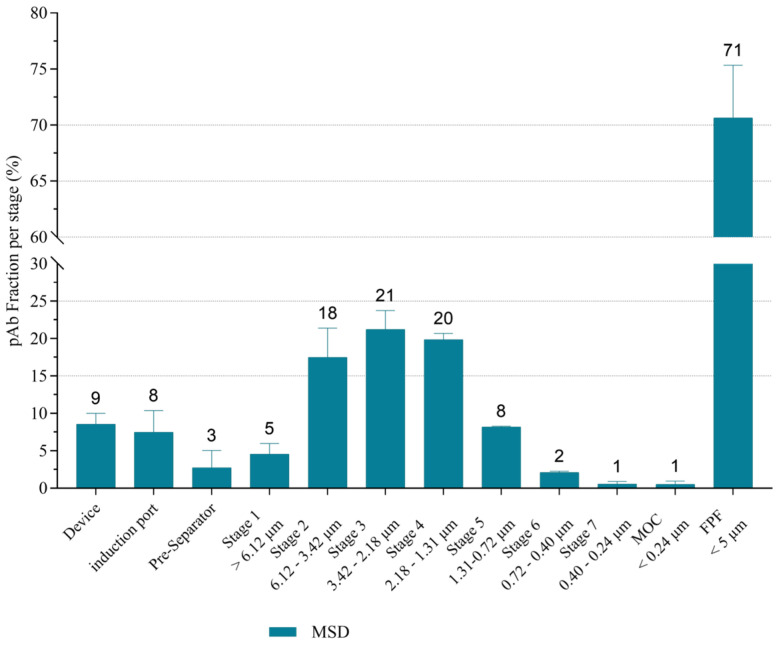
Aerodynamic deposition profiles of the DPIs based on D-mannitol or on D-mannitol/D-sucrose/Dextran 10 kda (MSD), mean ± standard deviation, *n* = 3. The evaluation was made at 100 L/min during 2.4 s using a next-generation impactor connected to a low resistance Axahaler^®^ device containing a hydroxypropyl methylcellulose capsule filled with 20 mg dried formulation, three capsules per test. Fine particle fraction is expressed relative to the nominal dose. Ind. Cut-off sizes are given for each stage.

The size distribution of the aerosol particles from the DPIs have shown a d(0.5) somewhat below 5 µm (between 4.4 ± 0.2 µm and 4.9 ± 0.6 µm for DPIs based on MSD or D, respectively, Figure 7) which is the upper limit for an appropriate lung deposition if the particle shape is close to a sphere and the density is close to 1. This result is similar to the particle size observed on the SEM pictures (Figure 3). The mean volume-to-weight diameters D[4,3] consider the aggregation state in DPIs. The lowest aggregated DPIs are those based on MD and MSD with a D[4,3] around 5 µm, which is significantly lower than those based on S or SD with a D[4,3] of 14.7± 5 µm and 16.5± 5.3 µm respectively; *p* ˂ 0.05, Tukey’s). Moreover D[4,3] of aerosols from DPIs based on MD and MSD is also lower than from DPIs based on M, D (i.e., 12.9 ± 0.1 and 11.7± 5.4 µm, respectively) or on SM (8.7 ± 0.8 µm), as reported in Figure 7. The largest aggregates from the aerosols are given by the d(0.9), which follows the same trend as D[4,3] (Figure 7). DPI based on SD presented the highest RM at T0 (Figure 4), which could favor cohesion and therefore aggregation, whereas DPI based on MSD present the lowest RM at T0 (13.0% versus 9.6%, respectively, Figure 4). Both d(0.9) and D[4,3] are found highest for DPI-SD and lowest for MSD. In terms of the percentage of particles inferior to 5 µm, which can be correlated with FPFn (Figure 8), the highest percentage was obtained for DPI-MSD, having the lowest RM at T0, but also with the lowest d(0.5) and D[4,3].

- Aerosol particle size distribution—Next generation impactor-based assessment.

As the produced DPIs aim to be delivered by inhalation, to treat pulmonary diseases, or even to be used as starting points for systemic administration of IgG by the pulmonary route, the aerodynamic behavior of a DPI included in the desirability region (i.e., DPI-MSD) was assessed. The efficiency of in vitro delivery was very high for DPI-MSD, with a fine particle fraction of 71 ± 5% (calculated from the nominal dose) very close to the % inferior to 5 µm obtained with laser diffraction (67 ± 4%) compared to, for example, DPI-M with a FPFn of 60 ± 5% (Figure 8) that was also very close to the % inferior to 5 µm obtained with laser diffraction (53.5 ± 0.7%). The highest fractions being deposed are between the stages 2 and 4 corresponding to aerodynamic diameters between 3.42 and 1.31 µm (Figure 8). The MMAD is 2.5 ± 0.2 µm and GSD is 1.80 ± 0.03. As GSD is below 2, the distribution is considered monodisperse. The total recovery of the assay was 93 ± 5%, relative to the dose in the capsule.

## 4. Discussion

For this proofsor-of-concept study, pAb were applied because of their cost-effectiveness and availability in large quantities. Unlike mAb, pAb are easier to purify and can be collected from bovine blood and milk without animal sacrifices [32,39]. Although degradation was only assessed via aggregation patterns, this remains a critical quality attribute in drug development and release testing [40]. The preliminary phase focused on the short-term stability of pAb, first in solution. Therefore, buffer systems were evaluated for their ability to maintain stability of pAb in solution. Buffers were chosen based on their pKa values to maintain the pH between 6 and 7, which is adequate for pulmonary delivery and minimizing degradation pathways that are more prevalent at acidic pH (i.e., unfolding, fragmentation and deamidation via hydrolysis) or at basic pH (i.e., unfolding, aggregation via dityrosine formation and deamidation via succinimide formation [10]. L-histidine (pKa = 6.0) [41], phosphate (pKa = 7.2) [42] and citrate (pKa = 6.4) [42] were tested. A citrate buffer at pH 7.0 showed the best performance, with 97 ± 3% of soluble protein recovery (i.e., low HOA content considered as negligible (3 ± 3%)) and high monomer recovery (100%, i.e., no LOA content, Figure 2). This is likely due to the chelating properties against metal ions [42], thereby mitigating metal-induced oxidation [43,44]. Moreover, pH 7.0 allows a high soluble pAb fraction compared to pH 6.0 with the pAb isoelectric point, which lies between 5.0 and 6.6. In contrast, L-histidine at pH 6.0 resulted in up to 20 ± 0.6% of HOA (Figure 2). The citrate buffer 20 mM at pH 7.0 was the buffer selected to produce the DPIs by spray-drying with sugar-derivative stabilizers.

The next phases focused on short-term (T0, i.e., just after spray-drying) and long-term pAb stabilities in DPIs generated by spray-drying pAb solutions with stabilizers, a polyol such as D-mannitol, a disaccharide such as D-sucrose and a polysaccharide such as dextran 10kDa, alone or in combination at a final pAb:excipient ratio of 9:1 *w*/*w*. The aimed advantages of generating a powder is to enhance long-term stability and to reduce transport and storage constraints [13,45].

Spray-drying was chosen, contrary to freeze-drying, for its ability to control particle size [14], essential for inhalation delivery. During spray-drying, proteins face four key stresses [21,46]: (i) high shear stress within the nozzle; (ii) high surface specific area between the solution and the air during the droplet formation; (iii) thermal stress; and (iv) dehydration stress.

Studies show that antibodies tolerate shear stresses well, while aggregation is mainly induced by air-liquid interfaces [47]. Despite a high inlet temperature (Tin) (i.e., 110 °C in this study), the outlet temperature remained moderate (i.e., between 55 and 60 °C) and exposure time is brief, i.e., a few seconds at most [21]. To mitigate dehydration stress and to improve stability during storage, sugar derivatives are used as excipients to form an amorphous glass matrix based powder [22]. They stabilize proteins via the glass matrix hypothesis, reducing global molecular mobility (α-relaxation, estimated by the factor τα) [22,48] and preserving structure during storage due to their rigid structure. Although not entirely correct, it is commonly assumed in pharmaceutical sciences that log(τα) scales correlate with the difference between the Tg and the storage temperature [48]. A second stabilization mechanism involves water replacement, in which excipients form H-bonds with protein surface residues [23], mimicking water’s role and stabilizing thermodynamically secondary structures, like the α-helix and β-sheets. However, this theory has been challenged, as some proteins lose their secondary structure upon drying, but regain full activity after rehydration [32,49]. Current understanding favors a dynamic stabilization model, where excipients reduce local molecular motions (β-relaxations) playing a more significant role in preserving protein integrity under dry conditions [32,48]. From this study, several trends emerged, despite the relatively good intrinsic resistance of the pAb to the spray-drying process and the use of a low stabilizer-to-protein mass ratio of 1:9. The best results just after spray drying (T0) were predicted for the combination of MS or MD at 64/4 or 38/61, showing the highest desirability value (Figure 6). However, similar desirability values are found for other compositions, including binary mixtures of D-mannitol and Dextran 10kDa in about equal fractions (desirability of 0.665 versus 0.670). During storage, the DPIs containing M with an about equal D fraction (55/45) and no S fraction showed the highest predicted desirability (0.748).

The conditions for lowest aggregation (LOA or HOA) were predicted as DPI-MD (51/49). This composition is about the same as the MD experiment in the mixture design (50/50), which indeed shows good acceptable results (desirability of 0.703 versus 0.704).

Several research groups have already explored why some sugars are better stabilizers than others. Smaller and molecularly more flexible saccharides stabilize some model proteins better than their larger and more rigid counterparts, probably by filling cavities better within the protein [46]. However, upon storage, the least stable forms evolved towards the thermodynamically most stable, which is the unfolded one. Newly, fully uncovered hydrophobic residues then form aggregates, probably upon reconstitution (in solution) [50]. These results suggest that even if both strategies were efficient, using excipients with diametrically opposed properties (i.e., one excipient with a very high Tg but suffering from topological constraints, such as a polysaccharide, and one excipient with a very low Tg, but with a high number of potential H-bonds and a high accessibility, such as D-mannitol) is better than combining one or the other with an excipient presenting an intermediate state (i.e., moderate Tg and moderate accessibility, such as D-sucrose).

In terms of HOA, the glass matrix seems to be predominant to stabilize pAb. Indeed, both DPI with Dextran 10 kDa (D and MSD) had no or hardly any HOA despite a higher level of LOA (5.2 ± 0.2% and 3.6 ± 1.2%, respectively), while the DPI with only D-sucrose had 4.07 ± 0.06% LOA and 3.0 ± 3.0% HOA (Table 2).

Regarding long-term stability, because the amorphous content and glass matrix state were identified as critical parameters, RM within the powders, after the DPI process, needs to be reduced as it may speed up the crystallization process and decrease the Tg. However, due to their hydration layer, which minimizes the protein free energy to participate in their structure maintenance, proteins tend to sequestrate water very tightly. A fully hydrated protein is estimated to contain up to 0.4 g H_2_O per gram of protein [51], while studies have shown that the energy of biological water in the vicinity of hydrophilic amino acids was 0.4 kcal/mol higher than that of bulk water, while once removed, it is replaced by another adjacent molecule in the pico- to nano-second scale [52].

It is thus not difficult to understand why RM remained so high (between 9.6% and 13.0% after spray-drying, Figure 4) even in the presence of water substitutes, such as polyols or sugars. However, such a quantity of water remains unusual for powders where the goal is to remove as much water as possible from the system. Some previous considerations in the literature talked about 1-8% to achieve a maximal dried protein stabilization [12], but this is expected to greatly vary from protein to protein and depends on the type of stabilizers.

RM can impact on the aerodynamic performance of DPIs by increasing the particle cohesion through increased capillary forces between particles, which would decrease the powder dispersion through the dry powder inhaler. DPI-MSD presents the lowest RM (9.6%) and the highest percentage of particles with a diameter below 5 µm, a d(0.5) close to the D[4.3], and the lowest d(0.9) (Figure 7), whereas DPI-SD showed the highest RM (13.0%), a significant lower percentage of particles with diameters below 5 µm (53 ± 6% versus 67 ± 4%, *p* ˂ 0.05, Tukey post hoc test), a significantly higher D[4.3] (16.5 ± 5.3 µm versus 4.5 ± 0.2 µm, *p* ˂ 0.05, Tukey post hoc test), whereas d(0.5) is similar (*p* ˃ 0.05) and d(0.9) is significantly higher (59 ± 26 versus 6.2 ± 0.3 µm, *p* ˂ 0.05, Tukey post hoc test) (Figure 7), which is in line with these hypotheses. The percentage of particles diameters inferior to 5 µm correlates well with the FPFn for the DPI based on M and MSD. Therefore, it seems that aerosol performance of pAB DPIs could be discriminated against with this fast and less sample-consuming technique (i.e., laser diffraction-based technique). DPI-MD showed promising aerosol performance with a percentage of particles with diameters inferior to 5 µm of 51 ± 3%, which is, however, significantly lower than the 67 ± 4% with DPI-MSD. This difference is not explained by a higher d(0.9) or D[4,3] due to RM, as is the case for DPI-S or DPI-SD (Figure 7). However, even though aerodynamic performances are important, the stability of the active drug is the most important to guarantee efficacy and safety. Considering these arguments, DPI-MD is the most promising DPI with low RM, low LOA and low HOA, and showing reasonable aerodynamic performance with a percentage of particles with diameter inferior to 5 µm around 50%, which is quite good in the inhalation field [46].

## 5. Conclusions

This study demonstrates that the stabilization of polyclonal immunoglobulin G (pAb) in dry powder formulations for inhalation is a multifactorial challenge, requiring a nuanced understanding of both protein behavior and excipient properties. The combination of D-mannitol and dextran 10kDa (MD) emerged as the most effective formulation, offering good protection against aggregation during spray-drying and long-term storage.

The ternary mixture, DPI also including D-sucrose (MSD), showed a promising short-term stability but was less effective than MD over time, highlighting the importance of excipient selection based on complementary physicochemical characteristics.

The findings support the dual role of excipients in protein stabilization: thermodynamic stabilization via hydrogen bonding and dynamic stabilization through reduced molecular mobility in the amorphous glass matrix. Residual moisture (RM) was identified as a critical parameter, influencing both protein integrity and powder performance, reinforcing the need for optimized drying and storage conditions.

From an aerodynamic point of view, the MD and MSD formulations exhibited favorable particle size distributions and fine particle fractions, confirming their suitability for deep lung deposition. These results underscore the potential of antibody-based dry powders for pulmonary delivery, not only for local treatment of respiratory diseases but also for systemic administration.

Future work should explore the scalability of these formulations, assess their immunogenicity and bioactivity in vivo, and investigate the applicability of these stabilizing strategies to monoclonal antibodies and other biologics.

## Figures and Tables

**Table 1:** Theoretical compositions and yield (%) of the spray-dried DPI formulations including the polyclonal antibody and sugar derivatives. DPI-MS is based on D-mannitol/D-sucrose; DPI-SD on D-sucrose/dextran 10 kDa; DPI-MD on D-mannitol/dextran 10 kDa; DPI-MSD on D-mannitol/D-sucrose/dextran 10 kDa. ^a^: percentage in relation to solid content; ^b^: sugar derivative percentage in relation to sugar derivative content.

DPI Type	Polyclonal IgG (%)	Buffer (%)	Mannitol (%)	D-Sucrose (%)	Dextran 10 kDa (%)	DPI Yield (%)
M	60.7 (90%) ^a^	32.5	6.8 (10%) ^a^ 100% ^b^	-	-	71.6
S	60.8 (90%) ^a^	32.3	-	6.8 (10%) ^a^ 100% ^b^	-	79.1
D	61.0 (90%) ^a^	32.2	-	-	6.8 (10%) ^a^ 100% ^b^	75.6
MS	60.7 (90%) ^a^	32.4	3.4 (5%) ^a^ 50% ^b^	3.4 (5%) ^a^ 50% ^b^	-	76.1
MD	60.8 (90%) ^a^	32.4	3.4 (5%) ^a^ 50% ^b^	-	3.4 (5%) ^a^ 50% ^b^	76.6
SD	60.7 (90%) ^a^	32.3	-	3.4 (5%) ^a^ 50% ^b^	3.4 (5%) ^a^ 50% ^b^	79.6
MSD	60.9 (90%) ^a^	32.3	2.3 (3.3%) ^a^ 33% ^b^	2.3 (3.3%) ^a^ 33% ^b^	2.3 (3.3%) ^a^ 33% ^b^	75.3

**Table 2:** High-order aggregate (HOA) and low-order of aggregate (LOA) percentages obtained for each DPI just after spray-drying (T0) and after 10 months in a desiccator at room temperature (T10), *n* = 3. * Considered as negligible.

DPI	HOA (%)		LOA (%)	
	T_0_	T_10_	T_0_	T_10_
S	7 ± 2	3 ± 3 *	4.0 ± 2.0	4.07 ± 0.06
M	1 ± 5 *	2 ± 5 *	2.3 ± 0.9	3.50 ± 0.60
D	1 ± 1 *	−2 ± 3 *	2.3 ± 0.4	5.20 ± 0.20
MS	3 ± 0	2 ± 3 *	1.6 ± 0.6	2.00 ± 0.30
SD	5 ± 2	−1 ± 2 *	0.8 ± 0.3	2.80 ± 0.30
MD	3 ± 3 *	−1 ± 2 *	0.2 ± 0.4 *	1.50 ± 0.30
MSD	2 ± 0	1 ± 1 *	−0.6 ± 0.6 *	3.60 ± 1.20

**Table 3:** Coefficient and results of ANOVA analysis on the quadratic model.

Response	Suggested Model	Coefficient D-Sucrose	Coefficient D-Mannitol	Coefficient Dextran 10 kDa	Significance for Quadratic Model	Exploitable (Lack of Fit NS)
T0 moisture	Quadratic	11.83	12.33	10.83	Yes (*p* = 0.0016)	Yes
T0 LOA	Quadratic	4.44	2.31	2.31	Yes (*p* < 0.0001)	Yes (*p* = 0.2617)
T0 HOA	Linear	NA	NA	NA	No (*p* = 0.0718)	No
T10 moisture	Quadratic	6.32	5.92	6.42	Yes (*p* < 0.0001)	Yes Robust
T10 LOA	Special cubic	4.16	3.29	5.29	Yes (*p* < 0.0001)	Yes with caution (*p* = 0.003)
T10 HOA	Linear	NA	NA	NA	No (*p* = 0.2017)	No

## Data Availability

The original contribution presented in this study is included in the article. Further inquiries can be directed to the corresponding authors.

## Abbreviations

The following abbreviations are used in this manuscript:

ANOVA	Analysis of variance
AUC	Area under the curve
BCA	Bicinchoninic acid
BEH	Bridged Ethylene Hybrid
BGG	Bovine gamma globulin
D	Dextran 10 kDa
Dgeo	Geometric diameter
DoE	Design of experiments
DPI	Dry powder for inhalation
DPI-MD	DPI including D-mannitol and dextran 10 kDa
DPI-SD	DPI including D-sucrose and dextran 10 kDa
DPI-MS	DPI including D-mannitol and D-sucrose
DPI-MSD	DPI including D-mannitol, D-sucrose, and dextran 10 kDa
FDA	Food and Drug Administration
FPFn	Fine particle fraction related to the nominal dose
GSD	Geometric standard deviation
H-bond	Hydrogen bond
HOA	High-order aggregates
LOA	Low-order aggregates
M	D-mannitol
mAbs	Monoclonal antibodies
MDSC	Modulated differential scanning calorimetry
MMAD	Median mass aerodynamic diameter
mw	Molecular weight
NGI	Next generation impactor
pAb	polyclonal IgG
PBS	Phosphate-buffered saline
RM	Residual moisture
RT	Room temperature
S	D-sucrose
SEC	Size exclusion chromatography
SEM	Scanning electron microscopy
T0	Time of analysis right after spray-drying
T6	Time of analysis after a 6-month storage
T10	Time of analysis after a 10-month storage
Tg	Glass transition temperature
XRPD	X-ray powder diffraction
